# Selinexor in combination with topotecan in patients with advanced or metastatic solid tumors: Results of an open-label, single-center, multi‐arm phase Ib study

**DOI:** 10.1007/s10637-021-01119-0

**Published:** 2021-04-28

**Authors:** Kyaw Zin Thein, Sarina A. Piha-Paul, Apostolia Tsimberidou, Daniel D. Karp, Filip Janku, Abdulrazzak Zarifa, Jatin Shah, Denái R. Milton, Stacie Bean, Lacey McQuinn, Jing Gong, Rivka Colen, Brett W. Carter, Vivek Subbiah, Deby C. Ogbonna, Shubham Pant, Funda Meric-Bernstam, Aung Naing

**Affiliations:** 1 Department of Investigational Cancer Therapeutics (Phase I Clinical Trials Program), Division of Cancer Medicine, The University of Texas MD Anderson Cancer Center, 1515 Holcombe Blvd, Houston, TX 77030 USA; 2 Division of Hematology and Medical Oncology, Oregon Health and Science University/ Knight Cancer Institute, Portland, OR USA; 3 Karyopharm Therapeutics, Newton, MA USA; 4 Department of Biostatistics, The University of Texas MD Anderson Cancer Center, Houston, TX USA; 5 Department of Diagnostic Radiology, The University of Texas MD Anderson Cancer Center, Houston, TX USA; 6 Department of Thoracic Imaging, Division of Diagnostic Imaging, The University of Texas MD Anderson Cancer Center, Houston, TX USA

**Keywords:** Selinexor, KPT 330, Topotecan, Metastatic solid tumors, Selective inhibitor of nuclear export (SINE)

## Abstract

*Background *Selinexor, a first-in-class, oral selective inhibitor of nuclear export (SINE) compound inhibits Exportin-1(XPO1), had demonstrated synergistic activity with many chemotherapies and conferred in vivo antitumor efficacy in hematologic as well as solid tumors. *Methods *This open-label, single-center, multi-arm phase 1b study used a standard 3 + 3 design and a “basket type” expansion. Selinexor with intravenous topotecan was given in one of the 13 parallel arms. Patients with advanced or metastatic relapsed/refractory solid tumors following prior systemic therapy, or in whom the addition of selinexor to standard chemotherapy deemed appropriate, were eligible. *Results *Fourteen patients with the median age of 61 years (range, 22–68years) were treated, and the most common cancer types were gynecological cancers; ovarian (*n* = 5), endometrial (*n* = 2), and 1 each with fallopian tube and vaginal cancers. Of the 14 patients treated, 12 (86 %) had at least one treatment-related adverse event (TRAE). The most common TRAEs were anemia (71 %), thrombocytopenia (57 %), hyponatremia (57 %), vomiting (57 %), fatigue (50 %), nausea (50 %), and neutropenia (36 %). Two patients had dose limiting toxicities. One patient dosed at selinexor 80 mg had grade 3 nausea and vomiting and one patient dosed at selinexor 60 mg experienced grade 4 neutropenia and thrombocytopenia. Of the 13 efficacy evaluable patients, one (8 %) with endometrial cancer achieved unconfirmed partial response (uPR) and the time-to-treatment failure (TTF) was 48 weeks, whereas 6 of the 13 (46 %) patients had stable disease (SD) contributing to the clinical benefit rate of 46 %. The median TTF for all patients was 9 weeks (range, 2–48weeks). *Conclusions *Once weekly selinexor in combination with topotecan was viable and showed some preliminary tumor efficacy. The recommend phase 2 dose of selinexor was 60 mg once weekly in combination with IV topotecan.

Trial registration: NCT02419495. Registered 14 April 2015, https://clinicaltrials.gov/ct2/show/NCT02419495

## Introduction

Cellular homeostasis maintains the intracellular localization of proteins via nuclear-cytoplasmic transport [1]. Cancer cells utilize this transport mechanism to elude anti-neoplastic therapies. Karyopherins are a group of proteins involved in the transport of macromolecules between the cytoplasm and the nucleus of a eukaryotic cell. There are at least seven nuclear export proteins, known as exportins, involved in the transportation of large molecules from the intranuclear compartment to the cytoplasm; hence regulating the cellular function to foster homeostasis [2]. Among those, exportin-1 (XPO1), or chromosomal region maintenance 1 (CRM1), is the most recognized exportin and XPO1 is responsible for the unidirectional export of ~ 220 different cargo proteins, including tumor suppressor proteins (TSP) and growth-regulating oncoproteins (GRP), from the nucleus to the cytoplasm [2–4]. This transport system is critical to normal cellular function, differentiation and development [4]. Altering the transport mechanism and upregulating the CRM1 was shown to be implicated in tumorigenesis in various malignancies [1, 5]. Moreover, XPO1/CRM1 overexpression has been associated with a negative prognosis in various cancers such as multiple myeloma, acute myeloid leukemia, malignant gliomas, pancreatic cancer and soft tissue sarcomas [1, 6–10] Exportin-1 has then become an attractive therapeutic target in cancer drug development by dysregulating the conveyance of regulatory proteins and thus leading to intranuclear accumulation of tumor suppressor proteins and inhibiting tumor growth.

Selective inhibitors of nuclear exports (SINE) were developed to modulate this synchrony by blocking the transport proteins, resulting in intracellular accumulation of TSP which restore intranuclear cell-cycle checkpoints, and halt the tumor growth entailing apoptosis of cancer cells [11–15]. Selinexor (KPT-330) is a first-in-class novel, oral potent inhibitor of XPO1 and was shown to inhibit tumor growth by blocking the nuclear-cytoplasmic transport mechanism and interfering DNA damage repair (DDR) mechanism in preclinical models [16–18]. Previous phase I and II studies had shown selinexor having modest activity as a single agent in solid tumors [19–21]. In vivo studies demonstrated that selinexor had synergistic activity with DNA damaging therapeutics, including chemotherapy, and potentiated cancer cell death when selinexor was combined with different chemotherapeutic agents in solid tumors [18, 22, 23]. To further investigate the safety, tolerability and clinical activity of selinexor in combination with standard therapies, we conducted an open-label, single-center, multi-arm phase IB trial of selinexor in combination with standard chemotherapy or immunotherapy agents in patients with advanced or metastatic solid tumors. Hereby, we are reporting results from the selinexor in combination with topotecan treatment arm. 

## Methods

### Patients

Eligible patients were 18 years and older with histologically documented, advanced or metastatic solid tumors (excluding brain tumors) whose disease did not respond to or had relapsed following prior systemic therapy or for whom the addition of selinexor to standard chemotherapy deemed appropriate and acceptable. Other key inclusion criteria included Eastern Cooperative Oncology Group (ECOG) performance status of 0 or 1 and adequate organ function. The number of prior treatments was not limited. Patients in the study had to have at least one measurable target lesion as defined by Response Evaluation Criteria in Solid Tumors (RECIST v1.1) [24, 25] for solid tumors, except for patients with castrate-resistant prostate cancer where Prostate Cancer Working Group 2 criteria were used [26]. Key exclusions were patients with primary central nervous system tumor or active central nervous system tumor involvement, evidence of complete or partial bowel obstruction or need for total parenteral nutrition, prior treatment with an agent targeting exportin, and unstable cardiovascular functions. The primary objective was to establish the safety and tolerability of selinexor when given in combination with standard chemotherapy or immunotherapy regimens, while secondary objectives included determining the disease control rate, objective tumor response rate, and progression-free survival of selinexor administered with standard chemotherapy or immunotherapy treatments. The primary efficacy parameter was the safety according to National Cancer Institute Common Terminology Criteria for Adverse Events (CTCAE) version 4.03 and the secondary parameters were clinical benefit rate (CBR; percentage of complete response [CR], partial response [PR] plus stable disease [SD]), disease control rate (DCR; percentage of CR, PR plus SD for at least 6 months, assessed according to RECIST 1.1 criteria), the objective tumor response rate (CR plus PR), assessed according to RECIST 1.1 criteria and progression-free survival (PFS) defined as the time between the cycle 1 start date and the date of disease progression or death, whichever is reported first. 

### Study design and treatment

This was an open-label, single-center, multi-arm phase IB of selinexor in combination with standard chemotherapy or immunotherapy treatments to determine the dose-limiting toxicities (DLTs) and maximum tolerated dose (MTD) of selinexor and further explore the safety and tolerability of the MTD in patients with advanced or metastatic solid tumors (ClinicalTrial.gov identifier: NCT02419495). The study was conducted in multi-arms utilizing a standard 3 + 3 design and a “basket type” tumor-specific expansion cohorts. The combination of selinexor and topotecan was evaluated in one of the arms. Selinexor was administered at either 60 mg twice a week (BIW) or 60–80 mg weekly (QW) in combination with topotecan 0.5 to 1.5 mg/m^2^ daily for 5 days in a 21-day cycle. The study protocol was approved by the Institutional Review Board or Independent Ethics Committee at MD Anderson Cancer Center and was conducted in accordance with the Declaration of Helsinki, Good Clinical Practice, and all local and federal regulatory guidelines. All patients signed informed consent prior to enrolling onto the study.

### Study assessments

Tumor response was assessed using RECIST v1.1. Baseline imaging was done within 30 days of treatment initiation. Repeat imaging (using the same methodology as at baseline) was obtained every 9 weeks. Treatment-emergent adverse events (TEAEs) and treatment-related adverse events (TRAEs) were graded using the CTCAE version 4.03. DLT was defined as any selinexor-related grade 4 hematologic adverse event, grade ≥ 3 thrombocytopenia associated with clinically significant bleeding, febrile neutropenia or non-hematologic adverse event ≥ grade 3 in severity per CTCATE (v 4.03) despite optimal supportive medications, excluding electrolyte abnormalities that are reversible, asymptomatic or hair loss which is not dose-limiting. The MTD was defined as the highest dose level at which ≤ 33 % of patients experience DLTs during cycle 1. After the MTD was defined in each schedule, the study was extended to include additional evaluable patients at the MTD. A safety monitoring committee comprised of investigators and the study sponsor reviewed all safety information and made consensus decisions about dose escalation.

### Statistical methods

Patient characteristics, TEAEs, TRAEs, tumor response, and time-to-treatment failure (TTF) were summarized using descriptive statistics. PFS time was computed from cycle 1 start date to the date of disease progression or death (if the patient died without disease progression), or the last evaluation date. Patients who were alive and did not experience progression of disease at the last follow-up date were administratively censored. Overall survival time (OS) was computed from cycle 1 start date to the last-known vital sign. Patients alive at the last follow-up date were administratively censored. The Kaplan-Meier method was used to estimate PFS and OS. All statistical analyses were performed using SAS 9.4 for Windows (Copyright © 2002–2012 by SAS Institute Inc., Cary, NC).

## Results

### Patient characteristics

A total of 14 patients with advanced, metastatic malignancies were enrolled between July 2015 and June 2017. Demographic and clinical characteristics of all patients enrolled are summarized in Table 1. The median age of patients was 61 years (range, 22–68 years). There was a substantial female preponderance with a female to male ratio of 3.7 :1 (79 % vs. 21 %). The median number of prior systemic therapies was 4 (range, 1–7). The most common cancer types were gynecological cancers; including ovarian (*n* = 5), endometrial (*n* = 2), and 1 each with fallopian tube and vaginal cancers and the rest included neuroendocrine cancer, malignant mesothelioma, desmoid fibromatosis, and colorectal cancer. Two patients were dosed at BIW dosing of selinexor while 6 patients received dose at 60 mg QW, and 6 patients were dosed at 80 mg QW. The median number of cycles completed for all patients was 2.5 (range, 0–11). For patients with SD, the median number of cycles completed was 4 (range, 2–7).

**Table 1:** Patients baseline demographics and disease characteristics

Characteristic	Topotecan 1.5 mg/m^2^ IV daily for 5 days Q3W	Topotecan 0.5 mg/m^2^ IV daily for 5 days Q3W		All patients (*N* = 14)
	Selinexor 60 mg PO BIW (*n* = 2)	Selinexor 60 mg PO QW (*n* = 6)	Selinexor 80 mg PO QW (*n* = 6)	
Age at consent (years)				
Median Range	49.8 (44.0-55.5)	61.2 (21.6–68.4)	63.2 (56.1–68.0)	60.5 (21.6–68.4)
Gender, *n* (%)				
Male	0	1 (17)	2 (33)	3 (21)
Female	2 (100)	5 (83)	4 (67)	11 (79)
Race, *n* (%)				
White	1 (50)	3 (50)	6 (100)	10 (71)
Hispanic	1 (50)	3 (50)	0	4 (29)
Black	0	0	0	0
Asian	0	0	0	0
ECOG performance status, *n* (%)				
0	1 (50)	0	0	1 (7)
1	1 (50)	6 (100)	6 (100)	13 (93)
Primary tumor, *n* (%)				
Ovarian	2 (100)	2 (33)	1 (17)	5 (36)
Breast	0	0	0	0
Colorectal Cancer	0	1 (17)	0	1 (7)
Endometrial/fallopian	0	1 (17)	2 (33)	3 (21)
Lung	0	0	0	0
Neuroendocrine	0	0	1 (17)	1 (7)
Pancreas	0	0	0	0
Esophageal		0	0	0
Head & Neck/salivary gland	0	0	0	0
Liver/cholangiocarcinoma	0	0	0	0
Sarcoma	0	0	1 (17)	1 (7)
Prostate	0	0	0	0
Others	0	2 (33)*	1 (17)**	3 (21)
Prior lines of systemic therapies, *n* (%)				
0–1	0	0	2 (33)	2 (14)
2–3	0	2 (33)	2 (33)	4 (29)
4–5	1 (50)	3 (50)	1 (17)	5 (36)
> 5	1 (50)	1 (17)	1 (17)	3 (21)

Abbreviations: BIW, twice weekly dosing schedule; ECOG, Eastern Cooperative Oncology Group; IV, intravenous; PO, oral; Q3W, 3 weekly dosing schedule; QW, weekly dosing schedule

* includes desmoid fibromatosis, and adenocarcinoma of unknown primary

** includes malignant mesothelioma (epitheliod type)

### Safety and tolerability

None of the 14 patients remained in the study and there was no expansion cohort for this treatment arm. Progression of disease accounted for the majority of patient withdrawals from the study and clinically unacceptable TEAEs contributed to withdrawal of two patients from the study. All 14 patients had at least one TEAE (100 %) whereas TEAEs related to selinexor were reported in 12 patients (86 %). The summaries of TEAE and TRAE are presented in Tables 2, 3 and 4. The most prevalent TEAE were anemia (86 %), fatigue (79 %), thrombocytopenia (71 %), hyperglycemia (71 %), hyponatremia (64 %), nausea (64 %), hypomagnesemia (57 %), and vomiting (57 %). The most common grade ≥ 3 TEAE were hyponatremia (29 %), anemia (21 %), neutropenia (21 %), thrombocytopenia (21 %) and leukopenia (14 %). TRAE in all grades of severity are described in Table 4. The most common TRAEs were anemia (71 %), thrombocytopenia (57 %), hyponatremia (57 %), vomiting (57 %), fatigue (50 %), nausea (50 %), neutropenia (36 %), leukopenia (29 %), and constipation (29 %). The most common grade 3/4 toxicities in all patients related to selinexor were hyponatremia (29 %), neutropenia (21 %), thrombocytopenia (14 %), leukopenia (14 %), and anemia (14 %). Two patients had dose limiting toxicities. One patient dosed at selinexor 80 mg had grade 3 nausea and vomiting and one patient dosed at selinexor 60 mg experienced grade 4 neutropenia and thrombocytopenia. Six patients (43 %) reported having serious adverse events (SAEs); 2 were considered related to study drug. One patient had treatment-related grade 3 anemia along with grade 2 pneumonitis and grade 3 dyspnea which were unrelated to treatment and one experienced grade 4 thrombocytopenia with grade 3 hyponatremia and acute renal insufficiency. Of the 4 patients with SAEs unrelated to study drug, one had grade 3 abdominal pain, one experienced grade 3 peritoneal infection requiring intravenous antibiotics and one patient had grade 3 laryngeal bleeding which required bronchoscopy with bronchial artery embolization. The fourth patient had grade 3 lung infection which ultimately led to grade 5 adult respiratory distress syndrome which was unrelated to study drug. One patient died with pneumonia during the study which was classified as unrelated to the treatment.

**Table 2:** Summary of bestoverall tumor response and time-to-treatment failure

Measure	All Patients (N = 14)
Response, n (%)	
CR	0
CR	0
PR*	1 (8)
SD	6 (46)
CBR (PR + SD)**	6 (46)
DCR (CR + PR + SD ≥ 6 months)	0
PD	6 (46)
Not evaluated	1
TTF in weeks, median (range)	
All patients	9 (2–48)
CBR patients	13 (8–22)
Number of cycles, median (range)	
All patients	2.5 (0–11)
CBR patients	4 (2–7)

Abbreviations: CBR, clinical benefit rate; CI, confidence interval; CR, complete

response; DCR, disease control rate; PD, progressive disease; PR, partial response;

SD, stable disease; TTF, time-to-treatment failure

* Unconfirmed PR (uPR) was observed in one patient with endometrial cancer

** Excludes uPR patient

**Table 3:** Summary of treatment emergent adverse events in the phase I safety population

Measure, n (%)	All Patients (N = 14)
≥ 1 TEAE	14 (100)
≥ 1 TRAE	12 (86)
Grade 3/4 TEAE	10 (71)
Grade 3/4 TRAE	8 (57)
SAE*	6 (43)
≥ 1 TRSAE*	2 (14)
At least one DLT**	2 (14)
Discontinued due to ≥ 1 TEAE	3 (21)

Abbreviations: DLT, dose limiting toxicity; SAE, serious adverse events; TRAE, treatment-related adverse events; TRSAE, treatment-related serious adverse events

**Two patients had dose limiting toxicities; one patient dosed at selinexor 80 mg had grade 3 nausea and vomiting and one patient dosed at selinexor 60 mg experienced grade 4 neutropenia and thrombocytopenia

*Six patients were reported to have SAEs; two were considered related to study drug. One patient had treatment-related grade 3 anemia along with grade 2 pneumonitis and grade 3 dyspnea which were unrelated to treatment. Another patient experienced grade 4 thrombocytopenia with grade 3 hyponatremia and acute renal insufficiency. Of the 4 SAEs which were unrelated to study drug, one had grade 3 abdominal pain, one experienced grade 3 peritoneal infection requiring intravenous antibiotics and one patient had laryngeal bleeding which required bronchoscopy with bronchial artery embolization. The fourth patient had grade 3 lung infection/pneumonia which ultimately led to grade 5 adult respiratory distress syndrome which was unrelated to study drug

**Table 4:** Summary of treatment-emergent and -related adverse events in all grades of severity

N (%)	Treatment-emergent adverse events (TEAE)		Treatment-related adverse events (TRAE)	
	All grades	Grade 3/4	All grades	Grade 3/4
Anemia	12 (86)	3 (21)	10 (71)	2 (14)
Leukopenia	5 (36)	2 (14)	4 (29)	2 (14)
Neutropenia	6 (43)	3 (21)	5 (36)	3 (21)
Thrombocytopenia	10 (71)	3 (21)	8 (57)	2 (14)
Constipation	7 (50)	0	4 (29)	0
Diarrhea	4 (29)	0	3 (21)	0
Nausea	9 (64)	1 (7)	7 (50)	1 (7)
Vomiting	8 (57)	1 (7)	8 (57)	1 (7)
Elevated AST/ALT	4 (29)	0	2 (14)	0
Elevated Alkaline phosphatase	5 (36)	0	3 (21)	0
Fatigue	11 (79)	1 (7)	7 (50)	1 (7)
Hyperglycemia	10 (71)	1 (7)	0	0
Hyperkalemia	4 (29)	1 (7)	2 (14)	0
Elevated lipase	3 (21)	0	1 (7)	0
Dehydration	3 (21)	0	3 (21)	0
Dyspnea	6 (43)	1 (7)	1 (7)	0
Cough	4 (29)	0	1 (7)	0
Hypomagnesemia	8 (57)	0	1 (7)	0
Hyponatremia	9 (64)	4 (29)	8 (57)	4 (29)
Hypoalbuminemia	4 (29)	0	2 (14)	0
Hypocalcemia	5 (36)	0	0	0
Hypokalemia	4 (29)	1 (7)	1 (7)	0

Abbreviations: ALT, alanine aminotransferase; AST, aspartate aminotransferase

### Antitumor activity

**Fig. 1:**
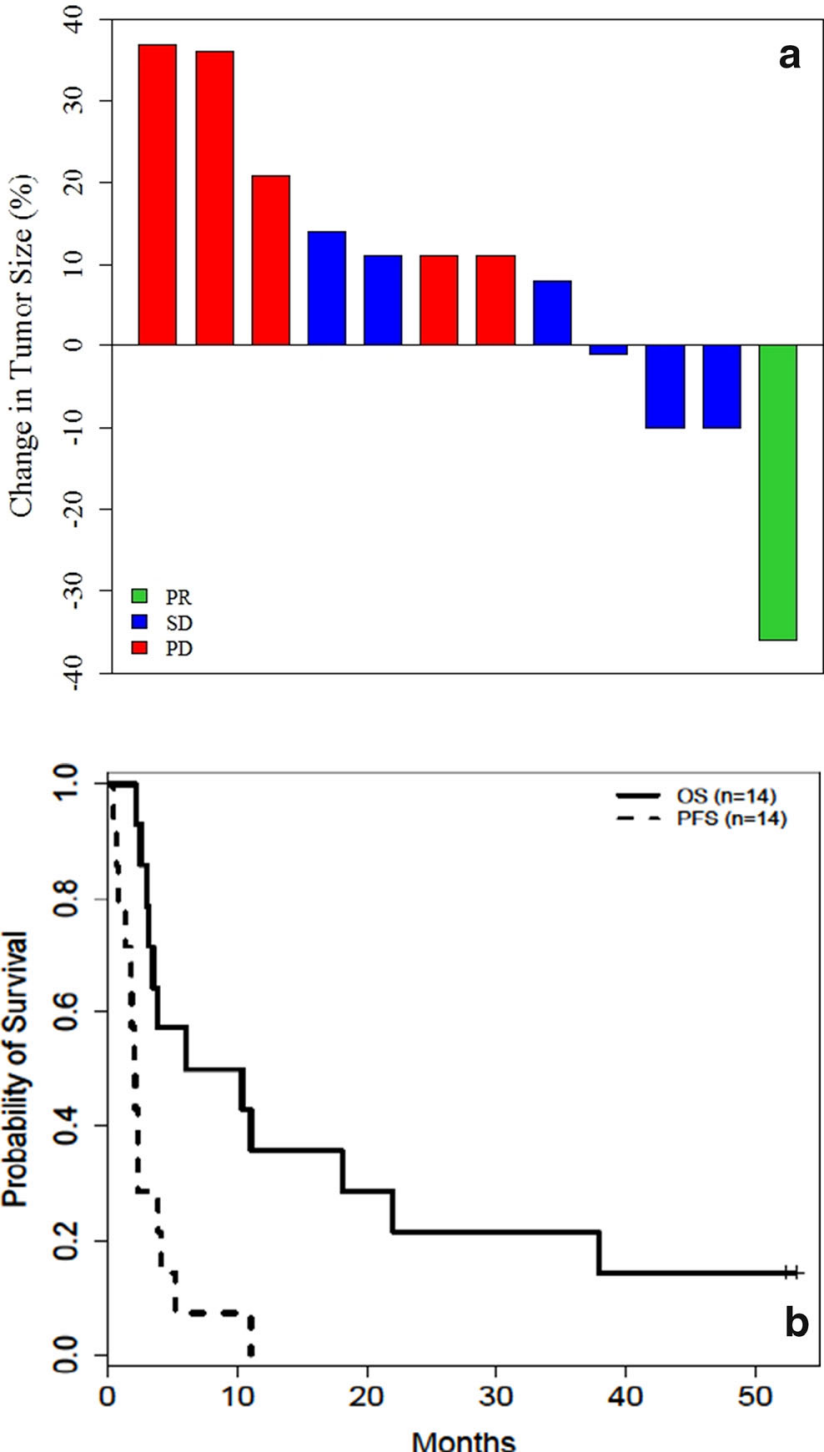
**a **Waterfall plot of maximum change in tumor measurements (per RECIST v1.1) for evaluable patients. **b** Kaplan-Meier plot showing progression free survival (PFS) and overall survival (OS) for all treated patients. Abbreviations: RECIST v1.1, response evaluation criteria in solid tumors version 1.1; PR, partial response; SD, stable disease; PD, progressive disease. *One PD patient had missing values for tumor change

Best overall tumor response is shown in Table 2. All 14 patients had measurable disease, but one patient had not completed their first restaging scans due to withdrawal of consent due to toxicity. Thus, 13 patients completed their first restaging scans per protocol and considered as efficacy evaluable patients. Per RECIST v1.1, a patient (8 %) with endometrioid adenocarcinoma who had progressed on prior 4 lines of therapies including platinum, taxol, gemcitabine and hormonal therapies, achieved unconfirmed PR and received a total of 11 cycles of treatments and the TTF was 48 weeks. Another patient with uterine carcinosarcoma who had 7 prior lines of therapies had SD with a TTF of 8 weeks. Four of the 5 patients with ovarian cancer were evaluable for efficacy. Two patients who progressed on 3 and 5 prior lines of therapies achieved SD with TTF of 9 and 17 weeks, respectively. No patient achieved CR in this treatment arm. Six patients (46 %) had SD contributing to the CBR of 46 %. None of these patients had SD for 6 months or longer; thus the disease control rate (DCR; percentage of complete response (CR) + PR + SD ≥ 6 months) was 0 %. The median TTF for all patients was 9 weeks (range, 2–48 weeks). For patients with SD, the median TTF was 13 weeks (range, 8–22). The median PFS and OS for all patients was 2.1 months (95 % confidence interval [CI]: 0.8, 3.9 months) and 8.2 months (95 % CI: 3.0, 22.0 months), respectively (Fig. 1b).

## Discussion

Our results showed that once weekly selinexor combined with topotecan was viable and showed clinical activity. Selinexor (KPT-330) is a first-in-class novel oral selective inhibitor of nuclear export (SINE) which irreversibly and covalently binds the cysteine 528 residue of the cargo-binding pocket [16, 17]. SINE was developed to inhibit tumor growth by blocking the nuclear-cytoplasmic transport mechanism (i.e., exportins 1–7), where the majority of bulky cargo proteins above 40kD including TSP and GRP, and many RNAs were transported through, and thus leading to accumulation of TSP/GRP, and apoptosis of cancer cells [1]. Moreover, topoisomerase I is a cargo of XPO1/CRM1 and one of the topoisomerase I resistance mechanisms is via XPO1 mediated nuclear exclusion of topoisomerase I, similar to topoisomerase 2 [27, 28]. Blockade of XPO1 leads to nuclear retention of topoisomerase I and DNA-topoisomerase I-topotecan complexes in the nucleus in turn causes apoptosis. A few phase I and II studies have shown modest activity of selinexor given as a single agent in patients with solid tumors [19–21]. Our group showed that selinexor had better efficacy and promising synergy when combined with different classes of chemotherapies; microtubule stabilizer or inhibitor, alkylating agent, topoisomerase inhibitor or even with pyrimidine analogue, in triple-negative breast cancer patient-derived xenografts [23]. Other preclinical studies have validated our finding that selinexor has synergistic activity with DNA damaging therapeutics; supporting a reasonable strategy to tackle solid tumors [18, 22, 23].

Shafique et al. reported their Moffitt Cancer Center experience of a phase II study of selinexor in patients with metastatic triple-negative breast cancer [19]. The efficacy of single agent selinexor was modest, with 3 of 10 (30 %) patients achieving SD for ≥ 12 weeks. The study was terminated early due to lack of objective responses [19]. In a phase IB study of selinexor in patients with refractory soft tissue or bone sarcoma, 30 of 52 (58 %) patients achieved SD while 17 (33 %) patients had durable response lasting more than 4 months; the antitumor activity was particularly noted in dedifferentiated liposarcoma [21]. Single agent selinexor was also studied in metastatic castration-resistant prostate cancer who were refractory to abiraterone and/or enzalutamide [29]. Although they showed some efficacy- 2 (25 %) patients achieving PR and 4 (50 %) patients with SD, 36 % experienced treatment-related grade 3/4 AEs and 21 % came off study due to intolerability. In another phase II study employing selinexor in gynecological cancers, single agent selinexor led to PR in 8 %, 9 %, and 4 % (DCR of 30 %, 35 and 24 %) in ovarian, endometrial and cervical cancers, respectively [20].

This arm of the open-label, single-center, multi-arm, standard 3 + 3 design with “basket type” expansion phase 1b study of selinexor in combination with standard chemotherapy in patients with advanced or metastatic solid tumors is the first study reporting selinexor in combination with topotecan, one of the standard chemotherapy regimens used in various tumor types. In our study, one patient with endometrioid adenocarcinoma (1/13; 8 %) achieved unconfirmed PR. This patient had progressed on 4 prior lines of therapies including platinum, taxol, gemcitabine and hormonal therapies, and the TTF was 48 weeks. No patient achieved CR. Six of the 13 (46 %) efficacy evaluable patients had SD, yet none achieved SD for 6 months or longer per protocol. Of the 5 patients with ovarian cancer, 4 were evaluable for response. Two of the patients who had progressed on 3 and 5 prior lines of therapies achieved SD with TTF of 9 and 17 weeks, respectively. The median TTF for all patients was 9 weeks (range, 2–48 weeks). For patients with SD, the median TTF was 13 weeks (range, 8–22).

All 14 patients had at least one TEAE whereas TEAEs related to selinexor (TRAE) were reported in the majority (86 %) of patients. The most prevalent TRAE and TEAE were hematological and gastrointestinal toxicities, fatigue, and electrolyte imbalance. Studies using selinexor monotherapy had previously shown that fatigue and hematological toxicities were the most common high-grade TRAE ranging from 6 to 21 %, while the most common grade ≥ 3 TRAE were hyponatremia (29 %), neutropenia (21 %), thrombocytopenia (14 %), leukopenia (14 %), and anemia (14 %) in our study employing selinexor in combination with topotecan. However, majority of patients (86 %) received once weekly selinexor dosing regimen in this study in contrast to prior single agent selinexor studies where twice weekly dosing regimens were implemented. Proper utility of growth factors and optimizing supportive care is crucial in this combination strategy.

Two patients had dose limiting toxicities. One patient dosed at selinexor 80 mg had grade 3 nausea and vomiting and one dosed at selinexor 60 mg experienced grade 4 neutropenia and thrombocytopenia. Six (43 %) patients reported having SAEs, 2 were considered related to study drug. One patient had treatment-related grade 3 anemia along with grade 2 pneumonitis and grade 3 dyspnea which were unrelated to treatment. Another patient experienced grade 4 thrombocytopenia with grade 3 hyponatremia and acute renal insufficiency. Of the 4 patients with SAEs unrelated to study drug, one had grade 3 abdominal pain, one experienced grade 3 peritoneal infection requiring intravenous antibiotics and one patient had laryngeal bleeding which required bronchoscopy with bronchial artery embolization. The fourth patient had grade 3 lung infection which ultimately led to grade 5 adult respiratory distress syndrome which was recognized as unrelated to study drug. One patient died with pneumonia during the study which was classified as unrelated to the treatment.

## Conclusions

Our study showed that once weekly selinexor in combination with topotecan was viable and showed clinical activity with a clinical benefit rate of 46 %. The recommend phase 2 dose of selinexor was 60 mg once weekly in combination with IV topotecan.

## Data Availability

The datasets used and/or analyzed during the current study are available from the corresponding author on reasonable request.

## Abbreviations

XPO1: exportin-1
CRM1: chromosomal region maintenance 1
TSP: tumor suppressor proteins
GRP: growth-regulating oncoproteins
SINE: selective inhibitors of nuclear exports
DDR: DNA damage repair
ECOG: eastern cooperative oncology group
RECIST v1.1: response evaluation criteria in solid tumorsversion 1.1
CTCAE: common terminology criteria for adverse events
CBR: clinical benefit rate
CR: complete response
PR: partial response
SD: stable disease
DCR: disease control rate
PFS: progression-free survival
DLT: dose-limiting toxicities
MTD: maximum tolerated dose
BIW: twice weekly
QW: weekly
TEAEs: treatment-emergent adverse events
TRAEs: treatment-related adverse events
TFT: treatment failure time
OS: overall survival
SAEs: serious adverse events
