# Correction to: Selinexor in combination with topotecan in patients with advanced or metastatic solid tumors: Results of an open-label, single-center, multi-arm phase Ib study

**DOI:** 10.1007/s10637-021-01192-5

**Published:** 2021-10-27

**Authors:** Kyaw Zin Thein, Sarina A. Piha-Paul, Apostolia Tsimberidou, Daniel D. Karp, Filip Janku, Abdulrazzak Zarifa, Jatin Shah, Denái R. Milton, Stacie Bean, Lacey McQuinn, Jing Gong, Rivka Colen, Brett W. Carter, Vivek Subbiah, Deby C. Ogbonna, Shubham Pant, Funda Meric-Bernstam, Aung Naing

**Affiliations:** 1 Department of Investigational Cancer Therapeutics (Phase I Clinical Trials Program), Division of Cancer Medicine, The University of Texas MD Anderson Cancer Center, 1515 Holcombe Blvd, Houston, TX 77030 USA; 2 Division of Hematology and Medical Oncology, Oregon Health and Science University/ Knight Cancer Institute, Portland, OR USA; 3 Karyopharm Therapeutics, Newton, MA USA; 4 Department of Biostatistics, The University of Texas MD Anderson Cancer Center, Houston, TX USA; 5 Department of Diagnostic Radiology, The University of Texas MD Anderson Cancer Center, Houston, TX USA; 6 Department of Thoracic Imaging, Division of Diagnostic Imaging, The University of Texas MD Anderson Cancer Center, Houston, TX USA

## Correction to: Invest New Drugs (2021) 39:1357–1365 10.1007/s10637-021-01119-0.

The article **Selinexor in combination with topotecan in patients with advanced or metastatic solid tumors: Results of an open-label, single-center, multi-arm phase Ib study** was originally published electronically on the publisher’s internet portal on 28 April 2021 without open access. After publication in volume [39], issue [5], pages [1357–1365] the author decided to opt for Open Choice and to make the article an Open Access publication. Therefore, the copyright of the article has been changed to © The Author(s) 2021 and the article is forthwith distributed under a Creative Commons Attribution 4.0 International License (https://creativecommons.org/licenses/by/4.0/), which permits use, sharing, adaptation, distribution and reproduction in any medium or format, as long as you give appropriate credit to the original author(s) and the source, provide a link to the Creative Commons licence, and indicate if changes were made.

The original article has been corrected.

